# Paediatric poisoning in rural Sri Lanka: an epidemiological study

**DOI:** 10.1186/s12889-018-6259-y

**Published:** 2018-12-06

**Authors:** Godakanda Arachchige Maneesha Prasadi, Fahim Mohamed, Lalith Senarathna, Rose Cairns, Pahala Hangidi Gedara Janaka Pushpakumara, Andrew Hamilton Dawson

**Affiliations:** 1 SACTRC, Faculty of Medicine, University of Peradeniya, Peradeniya, Sri Lanka; 2 Department of Pharmacy, Faculty of Allied Health Sciences, University of Peradeniya, Peradeniya, Sri Lanka; 3 TACT, Discipline of Pharmacology, Sydney Medical School, University of Sydney, Sydney, Australia; 4 Department of Health Promotion, Faculty of Applied Sciences, Rajarata University of Sri Lanka, Anuradhapura, Sri Lanka; 5 South Asian Clinical Toxicology Research Collaboration, Faculty of Medicine, University of Peradeniya, Peradeniya, Sri Lanka; 6 Discipline of Pharmacology, Sydney Medical School, The University of Sydney, Sydney, Australia; 7 New South Wales Poisons Information Centre, The Children’s Hospital at Westmead, Sydney, Australia; 8 Department of Family Medicine, Faculty of Medicine and Allied Sciences, Rajarata University of Sri Lanka, Anuradhapura, Sri Lanka; 9 Central Clinical School, University of Sydney, Sydney, Australia

**Keywords:** Paediatric, Acute, Poisoning

## Abstract

**Background:**

Acute paediatric poisoning is a common public health concern for both developed and developing countries. The type of agent and underlying cause differ depending on the social, cultural, economic and educational background. The objectives of this study were to identify the incidence and pattern of paediatric poisoning in a rural district in Sri Lanka and establish whether tertiary referral hospital data are a useful surrogate for estimating district level epidemiology of paediatric poisoning.

**Methods:**

A subset of epidemiological data were obtained from March 2011 to February 2013 from a randomized controlled trial (SLCTR/2010/008) conducted in 45 hospitals in Kurunegala district.

**Results:**

The age adjusted annual incidence of all cause of acute poisoning in children aged 1 to 12 years in the study area was 60.4 per 100,000. The incidence of poisoning of younger age group (1 to 6 years; 76 per 100,000) was significantly higher than older age group (7 to 12 years; 41 per 100,000) (*p* = 0.0001) in Kurunegala district. The annual incidence rate of paediatric admissions due to deliberate self-poisoning is 18 per 100,000 population. This study also established that admission data from primary hospitals provided the most accurate epidemiological information on paediatric poisoning.

**Conclusions:**

In rural districts of Sri Lanka, acute paediatric poisoning cases were less frequent and less severe compared to adult poisoning cases (426–446 per 100,000 population). The incidence of poisoning was significantly higher among young children with compared to old children. In this study, deliberate self-poisoning among older children was more frequently seen than in other comparable countries. Because most of the admissions are directed to and managed by primary hospitals, data from referral hospitals alone cannot be used to represent the true incidence of acute poisoning within a district. The data set from all the primary hospitals (*n* = 44) yielded more accurate poisoning incidence amongst a paediatric population.

## Background

Acute poisoning represents one of the most common medical emergencies in childhood [1–4]. However, there is a substantial under-recording and under-reporting of childhood poisoning incidents. This is due to multiple reasons including paediatric poisoning not being considered a notifiable condition, lack of reporting despite the presence of surveillance systems, misdiagnosis and patients/guardians not seeking medical treatment [2].

The type of agent and underlying cause of paediatric poisonings differs depending on social, cultural, economic and educational background [5, 6]. In developing countries, household products contribute to the majority of paediatric poisoning hospital presentations. In contrast, pharmaceuticals more commonly result in hospital presentations in developed countries [3, 7–11]. Some studies have shown that hospital admission rates for unintentional poisonings are higher in rural compared to urban areas [4, 12–14]. Conversely, other studies reported more acute paediatric poisoning admissions from urban areas [8, 15]. The incidence rate of adolescent poisoning has increased in recent years while the reduction in hospitalization of preschool children due to unintentional poisoning has also been reported [10, 14, 16–18].

Acute childhood poisoning in rural areas of lower-middle income countries is often under-reported, as most published data are obtained from urban areas [5, 15, 19]. Hospital referral/transfer patterns can affect data capture and toxicovigilance signals. In Sri Lanka, the majority of severely poisoned patients presenting to primary hospitals are transferred to rural tertiary hospitals. Thus tertiary hospital data can provide useful signals for severe poisoning. Conversely, low toxicity exposures are unlikely to be transferred, and thus tertiary hospital data will underestimate district or provincial poisoning rates for less-severe poisonings [11, 20, 21]. Furthermore, existing surveillance systems rarely link patients between primary and referral hospitals and in some cases results in double counting of the same patient in regional statistical data. This results in incorrect estimations of case fatality and morbidity rates [22, 23].

## Methods

The objectives of this study are to describe the epidemiology of paediatric poisoning in Sri Lanka in an entire rural district and determine whether tertiary referral hospital data are a useful surrogate in estimating epidemiology of paediatric poisoning for the district.

This study was a secondary analysis of data from a cluster randomized controlled trial (SLCTR/2010/008) of an outreach and centralized educational intervention promoting poisoning treatment guidelines to peripheral hospital staff members in North Western Province of Sri Lanka.

Paediatric patients in Sri Lanka are considered as children on or below 12 years old since the Ministry of Health, Nutrition and Indigenous medicine, Sri Lanka specified the maximum age limit for paediatric care as 12 years old [24]. Patients who are over 12 years of age are managed in adult wards. Further, the epidemiology of poisoning in patients who are over 12 years of age is predominately intentional self-harm.

North Western Province consists of the Kurunegala and Puttlalam district, and the Kurunegala district accounts for 70% of the population from North Western Province. This district had a total mid-year population of 1,618,465 with 95% residing rurally. Based on the 2012 census, 22% of the population was less than 12 years old in the North Western Province [25]. Data was collected from all 45 hospitals in Kurunegala district (44 primary hospitals and 1 tertiary referral centre). All patients (both adults and paediatric) who were admitted to these hospitals with a history of acute accidental or intentional poisoning were included in the study. From this, data from a paediatric population was obtained to assess the epidemiology of paediatric poisoning in Kurunegala district, Sri Lanka.

### Data collection

Trained research assistants visited primary hospitals (*n* = 44) once a month and traced acute poisoning admissions of previous month from the hospital admission book. Then patients’ details of traced admissions were collected from the hospital record room and the medical record scanned. Data including circumstances of exposure, clinical assessments, treatments and outcome details were recorded by medical staff. Data were extracted from patient’s record and then entered into a Microsoft Access database. Data from the referral hospital (*n* = 1) was collected prospectively as a part of an ongoing observational cohort study and entered into a cohort database by research assistants. Both these databases include all poisoning (intentional and unintentional) among all age groups.

Identification of the poisoning was based on different methods as described elsewhere [26, 27]. If the patient was poisoned with agrochemicals, chemicals or medicines, typically most of the guardians presented with the substances containers or packages. If the patient ingested plants, some guardians verbally confirm the ingested plants and some of the guardians brought the parts of the plants.

A subset of data was extracted from the primary hospital and the cohort database to identify paediatric patients (age ≤ 12 years) admitted following acute poisoning (from March 2011 to February 2013). Socio-demographic data (age, gender); exposure data (agents ingested/exposed, and circumstance of poisoning exposure); clinical details (symptoms, decontamination, use of antidotes); and outcomes (survival/fatality) were extracted. Identification of patients transferred from primary hospitals to the referral hospitals was done by using a data linkage computer algorithm to search the primary and referral hospital’s study database. Data linkage was done by matching transfer dates and admission dates, name, age, gender and poisoning type. In the case of algorithm failing, manual checking was performed.

### Statistical analysis

The annual midyear-populations on or below 12 years of age in 2012 Kurunegala district was used as the denominator in order to compute the paediatric population incidence of hospitalized poisoning. There were 349,115 children in the 0- to 12-year age group within the Kurunegala district.

“Observed” paediatric hospitalized poisoning incidence was calculated using three different possible admission datasets (outlined below). This was compared to what is considered the most accurate method as described by Senarathna et al., 2012, namely, the sum of all admissions to primary hospitals and to the referral hospital, minus all inter-hospital transfers [21].

The three data sets were:

i) All admissions to primary hospitals only;
ii) All admissions to the referral hospital only, both direct and referred from a primary hospital;
iii) The sum of all recorded admissions to primary hospitals and to the referral hospital (“all admissions”, the routine method used for government statistics, which counts referrals twice).

Summary statistics were used to describe different variables. Mean, median, interquartile ranges and proportions were used for descriptive statistics. Proportion comparison was used to examine the pattern of poisoning among gender and age groups. 95% confidence intervals (CI) were calculated for differences in proportions where indicated. MedCalc version 15.11.4 was used to compare two rates of patients according to the age adjusted incidence.

### Ethical approval

This randomized control trial was approved by the Ethical Review Committees of, Faculty of Medicine, University of Peradeniya, Sri Lanka (EC/2007/98) and Human Resource Ethics Committee, University of New South Wales, Australia (HREC 10129).

## Results

### Incidence of poisoning

During the 24 month study period, 422 children were admitted to all hospitals in Kurunegala district. Age adjusted annual incidence of poisoning in the study area was 60.4 per 100,000 inhabitants from 1 to 12 years of age and there were no patients aged under 1 year old. Of all patients, 69% were less than 6 years of age.

**Fig. 1:**
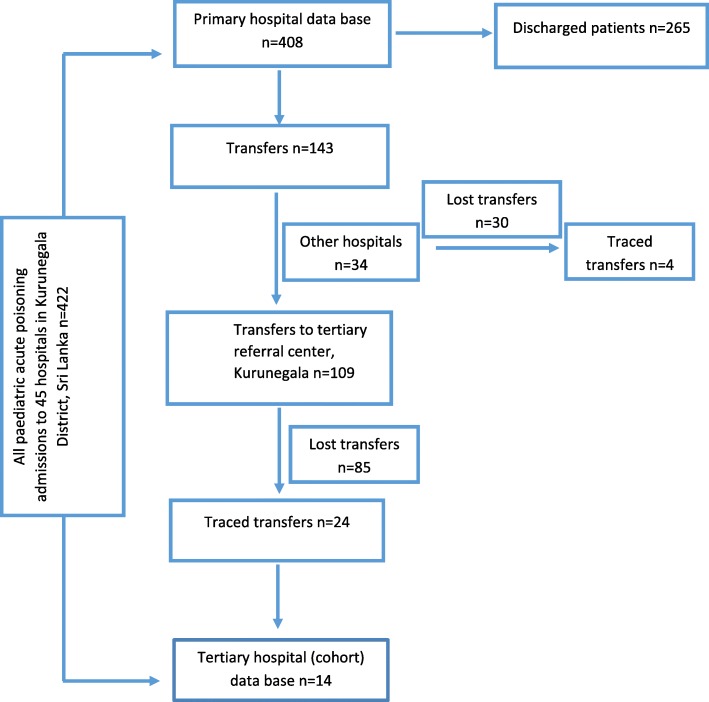
Paediatric acute poisoning hospital admissions in primary and tertiary hospitals in Kurunegala district, Sri Lanka

Of the paediatric patients admitted for acute poisoning, 408 (97%) were initially admitted to primary hospitals, and 143(35%) were subsequently referred for transfer to the tertiary referral hospital (*n* = 109, 26%), to base and district hospitals in the Kurunegala district (*n* = 21, 5%) and to other referral centers out of the district (*n* = 13, 3%). Only 6 patients were directly admitted to the tertiary referral hospital and only 28 transfers (20%) from primary hospitals to the referral hospitals (24 patients to tertiary referral centre and 4 patients to base and district hospitals in Kurunegala district) were traceable by the data linkage algorithm and manual checking (Fig. 1).

There were 8 transferred patients in the cohort database who were transferred from primary hospitals in Kurunegala district to teaching hospital Kurunegala, however, those patients were unable to link with primary hospital data. Therefore these 8 patients were included in all admissions to tertiary referral hospitals (Table 1) Thus, in total there were only 38 admissions (admissions in cohort data base+ traced transfers) to the tertiary referral hospital in the Kurunegala district (Table 1).

**Table 1:** Comparison of age adjusted annual incidence of poisoning based on three different data sets

	All admissions to primary hospitals only	All admissions to tertiary referral hospital only	All admissions to primary and tertiary referral hospitals
Number of patients	408	38	446
Age adjusted annual incidence of poisoning	58.4 per 100,000	5.4 per 100,000	63.8 per 100,000

As a consequence of the low numbers of paediatric poisoning (both direct and transferred) admissions to the rural referral hospitals, data from these hospitals alone grossly underestimates the provincial incidence of paediatric poisoning. The best estimate of the annual incidence of age adjusted paediatric poisoning is provided by data from primary hospitals (Table 1).

Age was not recorded for 2 male patients and these patients were not included in age-group comparisons. Age of children included in the study ranged from 1 to 12 years with a median age of 4 years (IQR 2–8).

### Age and gender variation

A similar number of females (*n* = 195) and males (*n* = 227) were admitted due to acute poisoning. The annual incidence of poisoning of the younger age group (1 to 6 years, median age = 3 years, IQR 2 to 4) was significantly higher (76 per 100,000 population) than older age group (7 to 12 years, median age = 10 years, IQR 8 to 12) (41 per 100,000 population) (*p* < 0.0001). Males (58%) significantly predominated in the younger age group (age 1 to 6-years, *P* = 0.006) while females (56%) were more represented in the older age group (age 7 to 12-years, *P* = 0.187).

**Fig. 2:**
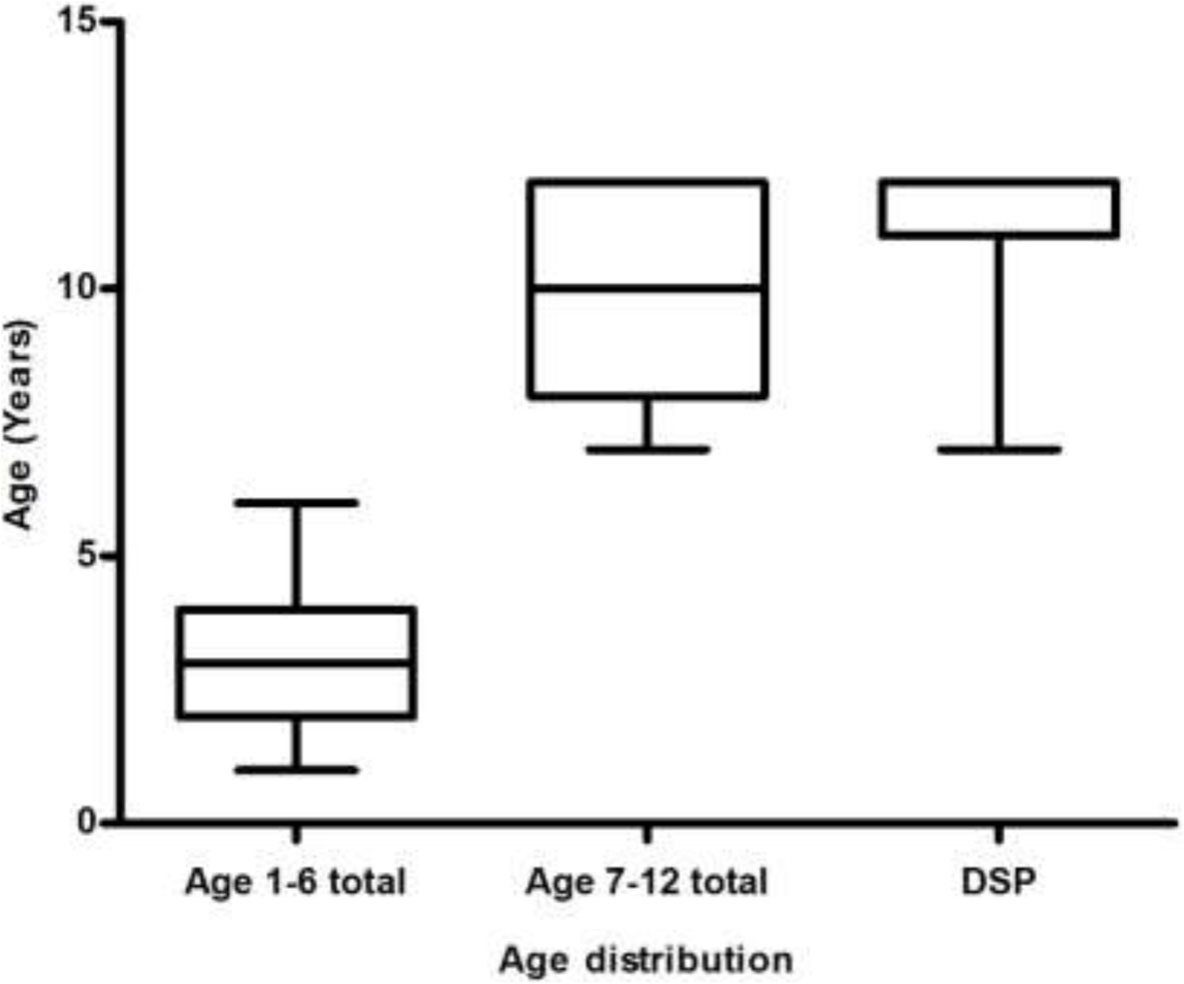
Age distribution of the acute poisoned paediatric patients admitted to hospitals in Kurunegala district (DSP = Deliberate self-poisoning)

All hospital admissions in the younger age group were due to accidental poisoning. In contrast,43% (*n* = 55) of the older children (7 to 12 years) were admitted due to deliberate self-poisoning and the annual incidence rate of paediatric admissions due to deliberate self-poisoning is 18 per 100,000 population. Furthermore, deliberate self-poisoning was significantly higher amongst females (*p* = 0.0001) and the median age of deliberate self-poisoning was 12 years (IQR 11 to 12) (Fig. 2).

### Types of poison ingested

Differing patterns of poisoning were observed between younger and older aged children (Table 2). Overall most of the admissions were due to plant poisoning (*n* = 123, 29%) followed by hydrocarbons (*n* = 91, 22%). We observed different patterns of poisoning between younger and older children. Hydrocarbons were the most common poisoning agents (*n* = 86, 30%) among young children, with kerosene (*n* = 71, 83%) the predominant hydrocarbon. Ricinus (*n* = 52, 73%) followed by oleander (*n* = 11, 15%) were the most common plant poisons accidentally ingested by younger children.

**Table 2:** Poison ingested by paediatric patients admitted to hospitals in North Western Province from March 2011 to February 2013

	Accidental poisoning		Deliberate self-poisoning n (%)	Unclassified N (%)
	Age 1 to 6 years n (%)	Age 7 to 12 years n (%)	Age 7 to 12 years n (%)	Age 7 to 12 years n (%)
Hydrocarbon	86 (20.3%)	2 (0.5%)	2 (0.5%)	1 (0.2%)
Plant	71 (16.8%)	24 (5.7%)	16 (3.8%)	12 (2.8%)
Medicines	58 (13.7%)	9 (2.1%)	22 (5.2%)	1 (0.2%)
Agro chemicals	29 (6.8%)	5 (1.2%)	10 (2.4%)	1 (0.2%)
Other chemicals	11 (2.6%)	2 (0.5%)	0 (0%)	0 (0%)
Unknown	36 (8.5%)	6 (1.4%)	5 (1.2%)	11 (2.6%)

The total number is equal to 420 since age of the two patients was missing

In contrast, the majority of the older children (age 7 to 12) ingested plants (40%) followed by pharmaceuticals (24%). Among deliberate self-poisoning patients, 40% of cases were due to pharmaceuticals (with paracetamol accounting for 35%of all self-poisoning cases) and 29% were due to plant poisoning (of which 22% was oleander poisoning).

### Poisoning outcome

During the study period, one death was reported due to pesticide poisoning and the fatality rate was 0.23% (95% CI, 0.00006–0.013203). The majority (49%) of the children were discharged and 34% were transferred from the peripheral hospitals to referrals centers for further medical care. The remaining 17% were discharged by their parents against medical advice.

Of the transfers, 66.4% of patients were aged between 1 to 6 years and the median age was 4 years (IQR 2–9). The majority (40.6%) of the transferred patients were poisoned with plants (ricinus (67%), oleander (22%), followed by hydrocarbons (17.5%) (Table 3). Among the deliberate self-poisoning patients, 24 (44%) were transferred to referral hospitals for further management.

**Table 3:** Characteristics of hospital transfers of acute paediatric poisoned patients

	Transfers which presented to tertiary hospital		Transfers which did not presented tertiary hospital		Total number of patients who referred to transfer n (%)
	Age 1 ≤ 6 children	Age7 ≤ 12 children	Age 1 ≤ 6 children	Age7 ≤ 12 children	
Hydrocarbons	2 (1.4)	0 (0)	22 (15.4)	1 (0.7)	25 (17.5)
Plants	3 (2.1)	0 (0)	33 (23.1)	22 (15.4)	58 (40.6)
Medicines	8 (5.6)	1 (0.7)	4 (2.8)	8 (5.6)	21 (14.7)
Agro c hemicals	6 (4.2)	4 (2.8)	7 (4.9)	4 (2.8)	21 (14.7)
Other chemicals	0 (0)	0 (0)	3 (2.1)	0 (0)	3 (2.1)
Unknown poisoning	0 (0)	0 (0)	7 (4.9)	8 (5.6)	15 (10.5)
Total	19 (13.3)	5 (3.5)	76 (53.1)	43 (30.1)	143 (100)

The proportion of patients who actually completed the transfer to the tertiary hospital following a decision in the primary hospital to refer was low (22%, (24/109). Majority of the completed transfers were medicines (38%) and agrochemical (29%) poisoned patients while 79% transfers belonged to the younger age group. Further results show only 7% (*n* = 4) deliberate self-poisoned patients arrived in the tertiary hospital.

## Discussion

This study provides detailed information on the complete epidemiology of acute pediatric poisoning hospital presentations in a rural district in Sri Lanka. It is likely that these findings are generalizable to other rural districts in Sri Lanka and could inform health planning and public health interventions. The overall age adjusted annual incidence of acute paediatric poisoning in the study area was 60.4 per 100,000 inhabitants from 1 to 12 years of age. The annual hospital presentation rate of paediatric poisoning patients is similar to study done in Queensland, Australia from 1998 to 1999 [13]. It is also considerably lower than the annual incidence of adult poisoning in Sri Lanka (426–446 per 100,000 population) [21, 23].

In this study, we found that the most accurate approach in providing a profile of paediatric poisoning within a district is from admissions in primary hospitals rather than rural tertiary referral hospitals. This is due to a higher number of admissions to primary hospitals and lower referral rates to tertiary hospitals. This is in marked contrast to higher risk adult poisoning cases where transfer rate from primary hospitals to referral centers is higher (70%) [21]. Hence, using data from referral hospitals for toxicovigilance and poisoning epidemiology is more reliable in the adult population. In contrast to our findings, studies conducted in Hong Kong and South Africa found that tertiary hospitals as a better source for representing the accurate value of paediatric poisoning admissions within the territories [28, 29].

The age adjusted incidence of acute paediatric poisoning presentation at hospitals was significantly higher amongst children aged 1 to 6 years compared with older children (*p* < 0.0001) in the Kurunegala district. All cases under the age of 6 years were due to accidental poisoning. This is consistent with both developed and developing countries that younger children are over- represented in acute accidental poisoning [3, 5, 11]. This is due to younger children being more inquisitive and exploratory in nature compounded by their newly acquired hand skills and mobility and increased the tendency to mouth objects [15, 30].

Self-poisoning among adolescence is common, however, among children under 12 years, it is rare and less frequent [13, 18, 25, 31]. In our study, there was a high percentage of hospital admissions due to deliberate self-poisoning (13% of all hospital admissions). In comparison, in other studies the percentage of deliberate self-poisoning in all hospital admissions ≤12 years ranged from 0.9–3.7% [4, 15, 32, 33]. Female (*n* = 38, 69%) admissions were significantly higher than male (*n* = 17, 31%) admissions (*P* = 0.0001) due to deliberate self-poisoning. This gender difference was also seen in epidemiological studies conducted in Lebanon, United Kingdom, Oxford, Singapore, Ontario and central Taiwan [16, 33–37]. Yet, other studies from India and the Czech Republic have described differing age and gender distributions of deliberate self-poisoning, with age between 9 to 13 years old and males predominating [32, 38].

The most common poisoning was ingestion of plant material accounting for 29%, of which 18% was due to Ricinus seeds. The majority of these cases were due to accidental ingestion. This differs from previously reported studies in the South Asian region from Sri Lanka, India, Pakistan in which kerosene was the most frequently ingested agent amongst a paediatric population [5, 8, 11, 15, 19]. This also differs from developed countries, whereby pharmaceuticals are the most common cause of paediatric poisoning hospital presentations [3, 39].

The commonest poisoning agent used for deliberate self-poisoning in this study was paracetamol (35%) followed by Oleander (22%). Similarly, a study done in Oxford reported that paracetamol was involved in the majority of self-poisonings in under 16-year-olds. In comparison, Lam found psychotropic drugs were more common among children between 10 and 14 years old. In India, the most common agent among self-poisoners ≤12 years old was rat poison [32, 40, 41].

The fatality rate of the paediatric poisoning was 0.2% during the study period and it is comparably lower than adult fatality rate in Sri Lanka [20, 21]. Previous Sri Lankan studies reported a 0.7% fatality rate in a paediatric referral hospital from 1985 to 2000 [42]. A retrospective study of poisoning admissions to urban and provincial hospitals in the western district in 1986 reported a 3.2% mortality rate [19, 42]. Recent studies conducted on plant and kerosene poisoning of children in Sri Lanka reported 0.3 and 1.2% case fatality rates [43, 44]. 0% case fatality rate was reported by other studies [11, 15, 45]. Case fatality rates in developed countries were ranged from 0.3 to 0.4% [13, 39].

Within the study cohort, 38% of the patients admitted to the peripheral hospitals were referred for transfer to the tertiary care units. Typically the patients were aged under 6 years old (71%) and plants were the most common exposure followed by hydrocarbons, medicines and agrochemicals. Referral for transfer was only slightly higher in patients with deliberate self-poisoning (44%). There may be a number of reasons for this apparent low rate. Local healthcare workers are aware that most plant and pharmaceutical poisonings are relatively benign and can be managed within the primary hospital context. As resources in primary hospitals are limited not all the patient received psychiatric consultation so there is limited incentive for the following transfer. [20, 43, 44].

In contrast to previous studies conducted on adult poisoning in Sri Lanka the rates of completed pediatric transfers were low [21]. This may reflect both perceived severity and the mode of transport. In our study patients with poisoning from agrochemicals and medicines are more likely to complete transfers and be transferred by ambulance. In contrast, many transfer referral would be for the parents to arrange transfer using private transport. This is expensive and time-consuming and it is likely that parents would be aware of the low potential for toxicity especially after the child had been assessed at the primary hospital.

Interventions including safe storage of toxic household products and pharmaceuticals, child-resistant packaging and educational interventions targeting different groups have been shown to be effective in reducing the incidence of poisoning [46–48]. These evidence-based interventions could be the focus on future public health strategies.

### Strengths and limitations

The main strength of this study is that we used data from all hospitals including primary and tertiary referral centres in Kurunegala district to calculate age adjusted annual incidence of acute paediatric poisoning.

However, there were few limitations of our study. We only used hospital presentation data and could not trace poisoning cases not presenting to the hospitals thus this underestimates the true community rate of paediatric poisoning. The majority of paediatric transfers could not be identified/matched. This is likely due to a number of factors, including the majority of them were done by private transport and thus parents may not have completed the transfers to tertiary referral centres and potential problems with the linkage algorithm.

## Conclusions

In rural districts of Sri Lanka, acute paediatric poisoning cases were less frequent and less severe than adult poisoning cases. However, deliberate self-poisoning among older children is not uncommon and there is evidence that it occurs far more frequently in the Sri Lankan community compared to other countries. Because most of the admissions are directed to and managed by primary hospitals, data from referral hospitals alone cannot be used to represent the true incidence of acute poisoning within a district. In comparison, data from primary hospitals capture more cases and thus provides a better estimate of paediatric poisoning incidence. The implementation of preventive and educational programmes will be crucial in order to reduce the number of severe intoxications, hospital admissions, and fatalities in children.

## Abbreviations

CI: Confidence intervals
DSP: Deliberate self-poisoning
IQR: Interquartile range
